# Using normalization process theory to evaluate the use of patient-centred outcome measures in specialist palliative home care—a qualitative interview study

**DOI:** 10.1186/s12904-023-01329-8

**Published:** 2024-01-03

**Authors:** Eva Lehmann-Emele, Maximiliane Jansky, Sabina Clapham, Susanne de Wolf-Linder, Claudia Bausewein, Farina Hodiamont

**Affiliations:** 1 Department of Palliative Medicine, LMU University Hospital, LMU Munich, Munich, Germany; 2 Department of Palliative Medicine, University Medical Center, Georg August University Goettingen, Goettingen, Germany; 3 Palliative Care Outcomes Collaboration, Australian Health Services Research Institute, University of Wollongong, Wollongong, NSW Australia; 4 School of Health Science, Institute of Nursing, Zurich University of Applied Sciences, Winterthur, Switzerland; 5 Wolfson Palliative Care Research Centre, Hull York Medical School, University of Hull, Hull, UK

**Keywords:** Specialist palliative home care, Patient-centred outcome measurement, Complexity, Qualitative research, Quality of care, Normalization process theory, Implementation

## Abstract

**Background:**

Standardised use of patient-centred outcome measures (PCOMs) improves aspects of quality of care. Normalization Process Theory (NPT) considers the social (inter-)actions of implementation processes operationalised through four constructs: *coherence-building, cognitive participation, collective action* and *reflexive monitoring*. The aim of the study was to identify barriers and enablers for the successful use of PCOMs in specialist palliative home care (SPHC) using NPT, to collect clinically meaningful and reliable data to improve patient outcomes.

**Methods:**

Qualitative study using semi-structured interviews with palliative care professionals from German SPHC teams who participated in a study using PCOMs. Data were analysed using Framework analysis, and contextualised within NPT.

**Results:**

Seventeen interviews across five teams were conducted. Some teams already had an understanding of what PCOMs are and how to use them, based on previous experience. In other teams, this understanding developed through the perception of the benefits (*coherence*). Participation and engagement depended on individuals and was decisive for coherence-building. The attitude of the management level also played a major role (*cognitive participation*). Integration of PCOMs into everyday clinical practice varied and depended on the manifestation of the first two constructs and other already established routines (*collective action*). In the context of appraisal, both positive (e.g. focus on patient) and negative aspects (e.g. additional work) of using PCOMs were mentioned (*reflexive monitoring*).

**Conclusions:**

Although benefits of using PCOMs were partly recognised, not all teams continued standardised use. Here, not only the social (inter-)actions, but also the influence of the context (working environment) were decisive. Future implementation strategies should consider integrating PCOMs in existing electronic patient records, education sessions supporting coherence-building, internal facilitators/local champions, and ensuring frequent data analyses as it is beneficial and increases the readiness of using PCOMs.

**Supplementary Information:**

The online version contains supplementary material available at 10.1186/s12904-023-01329-8.

## Background

Outcome measurement has become increasingly important in clinical palliative care. It delivers relevant information about the condition and situation of patients and relatives, providing a system to evaluate and improve palliative care. Measuring outcomes is not only helpful to identify needs, monitor symptoms and take clinical action, their use also enhances communication between clinicians and patients [1–5]. Preferably, outcomes are measured by standardised and validated questionnaires, that are self-reported by the patient and in which they assess their state of health (wellbeing and functional status) and quality of life in response to individual questions. [6, 7] However, in palliative care the use of patient-reported outcome measures (PROMs) can be challenging as patients often become increasingly unwell and are in many cases not able to self-report their symptoms, needs, and wishes due to high symptom/problem burden, cognitive impairment, or unconsciousness. To avoid exclusion of these vulnerable patient groups, patient-centred outcome measures (PCOMs), which also focus primarily on aspects that are important for patients and their relatives, can be used with the possibility of proxy-reporting by, for example, healthcare professionals [1, 7, 8].

In Australia, the Palliative Care Outcome Collaboration (PCOC) is a national program funded by the Australian government to enable outcome measurement and benchmarking [9]. In addition, Australia has an established funding model for palliative care based on case-mix [10–12]. In the UK, various initiatives, e.g. the Outcome Assessment and Complexity Collaboration (OACC) [13] or RESOLVE also aim to implement outcome measurement [8]. Although using outcome measures improves the quality of palliative care and is necessary for comparisons across services, the implementation of outcome measures is not yet established in a standardised and comprehensive way in specialist palliative care [14]. Barriers described include, for example, lack of knowledge about benefits, lack of training for users, lack of role models to encourage use, or fear of additional work and resulting lack of time [15, 16].

In Germany, the nationwide research project COMPANION (development of a patient-centred complexity and casemix classification for adult palliative care patients based on needs and resource use) used PCOMs in three specialist palliative care settings (palliative care unit, hospital advisory team and specialist palliative home care (SPHC)). The aim of the COMPANION project was to develop a German complexity and casemix classification for palliative care patients based on needs and resource use [17]. Its application will require the continuous and comprehensive use of PCOMs by healthcare professionals. Therefore, an implementation strategy tailored to the working environment of specialist palliative care needs to be applied, based on in-depth understanding of the processes behind. To facilitate this, it is necessary to assess, within a theoretical construct, the users' perspective on how the standardised use of PCOMs can be realised [18].

Normalization Process Theory (NPT) according to May and Finch (2009) is a sociological behavioural theory describing how likely it is that new practices, in this case using PCOMs, will be successfully adopted based on the characteristics of four interrelated constructs. It aims to theorise the process of how innovations (using PCOMs) become routines in everyday work and are thus normalised. NPT focuses on social (inter-)actions and thus on work that needs to be done by individuals or groups involved. These social (inter-)actions are operationalised through four constructs: *coherence-building*, *cognitive participation*, *collective action* and *reflexive monitoring* (see Table 2) [18, 19]. McNaughton et al. described these constructs in their qualitative study as ‘making sense of it’, ‘working out participation’, ‘doing it’, and ‘reflecting on it’ to make them more usable [20]. Furthermore, May and Finch locate the four constructs in the Context-Mechanism-Outcome (CMO) approach of ‘Realist Evaluation’, which is based on the assumption that the implementation context (environment teams operate in) influences the (inter-)actions (mechanisms) and thus the outcome (effects) of implementation work. The inclusion of the context which might be enabling or not, and how this may interact with the mechanisms to achieve a particular outcome, is considered as important [21].

Due to various regulatory structures and processes in German specialist palliative care, even services in the same setting can be heterogeneous, which is especially the case in SPHC [22, 23]. To address this, we focused on evaluating the use of PCOMS in the SPHC setting across different federal states. Therefore, the aim of the study was to identify barriers and enablers for the successful use of PCOMs in SPHC using NPT, to collect clinically meaningful and reliable data to improve patient outcomes. Objectives were to explore attitudes and experiences of staff towards the understanding, relevance and applicability of PCOMs in everyday SPHC.

## Methods

### Design

Cross-sectional multi-centre qualitative study using semi-structured interviews. This paper has been written in accordance with the COREQ checklist (Additional file 1) [24].

### Setting and participants

The COMPANION project collected data (between 01/2021 and 09/2022) on patients’ complexity documented electronically by healthcare professionals, if possible integrated in the existing electronic patient record (four teams integrated, two teams not integrated), over a three-month time period using several PCOMs (Additional file 2) [17]. Six SPHC teams participated in the COMPANION study, located in five different federal states across Germany. Teams were offered two education sessions by the COMPANION research team. Assessment was supported by pocket cards (describing how to use the measures) and a more in-depth manual describing the procedure for using the PCOMs in daily routine. Within the electronic documentation of PCOMs, it was possible to illustrate the assessed parameters in graphs. All teams had several monitoring meetings throughout data collection in the COMPANION study and received a final report on the analysis of collected patient outcomes presented by the research team.

For this qualitative study, which was conducted as an add-on in teams that participated in the COMPANION study, interview-participants, who used PCOMs to collect data on patients’ complexity, were recruited via phone or email by ELE. To cover a variety of views and experiences, and because of limited resources, purposive and convenience sampling was applied [25]. The sampling frame included professional groups (doctors, nursing staff and allied health professionals), management position, professional experience, age and gender (Additional file 3). Interviews took place approximately four weeks after the end of data collection of the COMPANION study to ensure that interviewees had sufficient time to gain experiences in using PCOMs in their daily routine, but that study-related experiences were still present. Interviews were conducted by ELE and FH by video call or phone as requested.

### Interview guide

Interviews followed a semi-structured interview guide (Additional file 4). Participants were encouraged to share their own personal experiences, attitudes and perspectives and to include opinions/views of their team members as well. It was developed according to Helfferich’s four-step procedure: collecting, reviewing, sorting, summarising [26], and is partly based on the interview guide of the study by Bradshaw et al. [14] as well as on experiences made during the COMPANION data collection. It was discussed in a multidisciplinary research group focusing on structure and clarity of questions. Two test interviews (doctor and nurse working in palliative hospital advisory care participating in COMPANION) were conducted to obtain further information about the appropriateness of questions, duration of the interview, and whether the NPT concept was covered by the interview questions. No changes were necessary.

### Data management and analysis

Interviews were audio recorded and transcribed verbatim, using transcription rules according to Dresing and Pehl; e.g. capital letters were used when the interviewees gave special emphasis to words [27]. The qualitative data management software MaxQDA 2022 (VERBI Software, 2021) was used to support analysis [28]. Data were analysed using content analysis, and by applying the framework method developed by Ritchie and Lewis [29]. The framework analysis aims to develop a systematising matrix which summarises/reduces the data material and thus allows for transparent comparison [30]. A recently developed NPT coding manual for qualitative research by May et al. was used for theoretical framing. It aims to facilitate transparency in data analysis processes and to simplify the theory for users [21]. The data was first analysed inductively to identify themes and to avoid exclusion of themes; therefore three interviews were double coded and compared by ELE and FH. Second, ELE developed the coding tree (Additional file 5) deductively based on the coding manual, as the inductively identified themes could be well assigned within the coding manual. Four interviews were double coded (ELE) for intra-coder reliability within two months apart. Both coding tree and manual were discussed multiple times in an interdisciplinary research group.

## Results

Overall, 17 interviewees were recruited in five participating teams (see Table 1). Teams were located in five different federal states across Germany, three teams in an urban area and two teams in a rural area. 17/20 persons accepted the invitation, one person did not respond at all. One team declined the interview request entirely due to lack of staff resources. Interviews lasted on average 29 min (range 13–55 min) and were conducted between January and August 2022. Median time between the end of the COMPANION data collection and the interviews was nine weeks (range between 4 and 16). Table 1 shows socio-demographic details. Within the participating teams, data saturation was reached as no new aspects were mentioned.

**Table 1:** Socio-demographic details of participants (*n* = 17)

		***n* = 17**
Profession	Doctors	8
	Nurses	8
	Psychologist/Social worker^a^	1
Position	Management level	10
	Non-management level	7
Work experience (years)	< 5	6
	5 – 10	4
	10 and more	7
Age group (years)	< 30	1
	31 – 45	6
	46 – 59	8
	60 and older	2
Sex	Female	13
	Male	4
Team	A	2
	B	3
	C	6
	D	3
	E	3

^a^The interviewee had both qualifications and a hybrid role in the team

All inductively developed codes could be verified within the NPT coding manual comprising 12 constructs, and corresponding to the Context-Mechanism-Outcome approach of Realist Evaluation [21]. Results are therefore presented as follows: Implementation Context, Implementation Mechanisms and Implementation Outcomes, which each are further divided into subcategories according to the NPT coding manual (see Table 2). Each section then describes how these (sub-)categories could be applied to the interview material.

**Table 2:** Main results based on the NPT coding manual [21] adapted to the application of PCOMs in SPHC

CMO Domain	NPT construct	Description and main results
**Implementation Context**	Strategic intentions	*How does the working environment affect the design and planning of the use of PCOMs?* Different approaches were recognized as three teams planned to further use PCOMs and two teams only wanted to participate in the study
	Adaptive execution	*How does the working environment affect the way in which users can find and implement ways of working that make the use of PCOMs an implementable project for practice?* Problems with software documentation in general were barriers and the integration of PCOMs into existing electronic patient record is crucial
	Negotiating capacity	*How does the context affect the extent to which the use of PCOMs can fit or be integrated into the existing ways of working of their users?* Internal communication and daily care organisation of teams were decisive for the extend of integration, as well as legal requirements and conditions
	Reframing organisational logics	*How do the existing social-structural and social-cognitive resources affect the implementation environment?* This is related to attitude of management level and therefore addressed under cognitive participation and initiation
**Implementation Mechanisms**	**Coherence-building** *How do people individually and collectively make sense of the use of PCOMs?*	
	Differentiation	*How do participants distinguish the use of PCOMs from their previous way of working?* The standardised, repeated outcome measurement framework was new for all participating teams
	Communal specification	*How do participants collectively achieve an understanding about the use of PCOMs?*
	Individual specification	*How do participants individually achieve an understanding about the use of PCOMs?* PCOMs were unfamiliar to use at first, but manageable after some time; difficulties in assessing psychosocial items/relatives and to achieve a consistent assessment by all team members occurred. In addition, contact in the SPHC setting is irregular which makes it more difficult to use PCOMs
	Internalisation	*Does the use of PCOMs make sense for the people involved?* Degree of internalisation was dependent on attitude of professionals and their recognition of benefits
	**Cognitive participation** *How do people engage to ensure that PCOMs can be applied?*	
	Initiation	*Which role does the leadership/key persons take on?* Different approaches were recognised, open communication and support of management level was most helpful
	Enrolment	*How do participants assess the introduction to the study and the training material and how was it used?* Support of research team (educations sessions, availability for specific questions) and provided training material was mainly considered helpful
	Legitimation	*How do participants come to believe that using PCOMs is right and should be part of their work?* Personal process of each individual, recognising own benefits was decisive
	Activation	*How do people involved support the use of PCOMs?* All teams had conversations about reliable use; internal facilitators/champions are most helpful for the process
	**Collective action** *How do people integrate PCOMs into their daily working practice?*	
	Interactional workability	*How do participants use PCOMs in their everyday work?* PCOMs were used for structuring patient conversations, content-related exchange within the team, deriving actions and evaluating their effectiveness, a quick overview of symptoms/problems, same language spoken, common attitude, simplified/reduced documentation, prioritising of care
	Relational integration	*How does the use of PCOMs affect the trust that participants have in each other?* Using PCOMs created a responsibility as colleagues relied on it within their own care
	Skill-set workability	*Is the work required to apply PCOMs allocated appropriately to those involved?* Assessing the PCOMs was divided according to expertise of the professionals
	Contextual integration	*Are resources made available for implementing the use of PCOMs?* Resources (working hours) were mostly made available for the application of PCOMs in daily care in all SPHC teams
	**Reflexive monitoring** *How do people individually and collectively appraise the use of PCOMs?*	
	Systematisation	*How do those involved have access to information about the impact of using PCOMs?* Information about the impact of using PCOMs was provided by the research team through feedbacks and a final report, which was considered positive, but further analyses by the teams themselves had not been done yet
	Communal appraisal	*How do participants evaluate the impact of using PCOMs?* Predominantly positive: using PCOMs increased the focus on the patient, symptom burden, and care system/relatives, shortened documentation and reading time, common language spoken, more structure in consultations, same data across settings, therefore avoiding information loss. Negative aspects were additional time burden and work
	Individual appraisal	*What further benefits/use of PCOMs can participants envision?* Using PCOMs as screening instruments for palliative care needs, enable comparisons between services, transparent data for third parties and involvement of relatives/employees in nursing homes
	Reconfiguration	*How do practitioners change their own work in response to their appraisal of using PCOMs?* Three teams changed documentation in line with the study, one integrated palliative care phases and one kept the original documentation
**Implementation Outcomes**	Intervention performance	*What practices have changed over time through the operationalisation, implementation and reproduction of the use of PCOMs?*^a^
	Relational restructuring	*In what ways has the use of PCOMs changed the way people are organised and relate to each other?*^a^
	Normative restructuring	*In which way has the use of PCOMs changed the norms, rules and resources that govern action?*^a^
	Sustainment (normalisation)	*In what way has the use of PCOMs become established in practice?*^a^

^a^This cannot be answered on the basis of the data material, as PCOMs have not been used long-term

### Implementation context

Implementation contexts deal with how the use of PCOMs is influenced by the environment the teams operate in, and consists of four further components. *Strategic intentions* of teams prior to the COMPANION study varied. Three teams wanted to use the study to test and possibly implement using PCOMs in their daily work, two teams had planned to only use them in the study from the beginning. Those two teams used a different software system for their daily documentation than the one used in the study. They therefore had to document twice, which had a negative impact on *adaptive execution*. Problems with software documentation were also mentioned as difficult in all teams, especially when loading was slow or over-savings happened through simultaneous documentation.

"We always do an initial assessment together, doctor and nurse. We are always consulting the patient together. With regards to the documentation, (…) there were a few difficulties, IT problems, who saves what and when? The data is directly IN there and you also have a baseline value. And THAT was a bit, yes, bumpy in the first phase, as it ALWAYS is. And then every team (doctor and nurse) had to readjust, right? In the first week, those were the challenges for me and I knew: OK, one team has somehow got it together in the first assessment, first one documents, THEN the other documents. (…) But with the NEXT team it didn’t work, because one had documented it before the other did and then the value was not in the basic assessment. So these were the very small problems that occurred at the beginning. Until everyone was THROUGH it, we had to find a way to make the documentation work.” (Doctor_No1_management-level, Pos. 30).

*Negotiating capacity* includes the way teams communicated and shared information, which impacts on usage and acceptance. PCOMs were perceived as helpful when teams conducted handovers text-based in digital form, and less helpful when they shared information in person or in weekly team meetings. Size of teams and staff fluctuation as well as the organisation of daily care routines also had an influence, e.g. whether teams were organised in a primary nursing system in which team members act as primary reference person for their patients and their relatives, or whether the staff member of reference changed daily.

“That's just the way it is, you don't see every patient, always just one and the same, but it changes both in the palliative care advisory service and on the ward as well as in the SPHC, to get this information from the previous health professional is, I think, extremely helpful.” (Doctor_No16_management-level, Pos. 40).

Furthermore, teams operate in different legal requirements and conditions, which relate to funding models, range of services, and staff-mix (teams consist of doctors and nurses only or include other professional groups, e.g. psychosocial). Two teams are independently organised and three are affiliated with a university hospital, which affected the hierarchical structures both between the management and the non-management level and between professional groups. Independently organised teams were described as less hierarchical, nurses felt more heard and communication in general tended to be more on eyelevel, which influenced how decisions regarding PCOMs were made. *Reframing organisational logics* focuses on existing social and social-cognitive resources. As this relates to coherence and attitude of the leadership/local facilitators, it is addressed under *cognitive participation* and *initiation*.

### Implementation mechanisms

#### Coherence-building

*Coherence-building* (‘making sense of it’) deals with how people collectively and individually make sense of using PCOMs and is further divided into four subcategories. For *differentiation*, some PCOMs (e.g. pain assessments, functional status) were already used more or less in every team, mainly in the context of initial assessment on admission, but the main information was documented in text/progress reports. A standardised assessment and response framework for implementation into daily routine had not yet been implemented in any of the teams, so the application of PCOMs differed significantly from how teams have worked before. Previous experiences play a role, which are explained in the categories *communal and individual specification*. They could not be distinguished in the data material, are therefore summarised, and refer to statements about how participants have used PCOMs previously and experiences they have had with their use. Overall, feedback was that the application was unfamiliar at the beginning, but interviewees quickly got used to it, as assessing the patient's situation is part of their daily work and developing new routines simply took some time.

“To be honest, I have to say that I was concerned before we introduced it, because I saw great difficulties in applying it to every patient, including telephone contacts, and that it would perhaps also exceed the time frame, so that we would have much less time for the patients. To be honest, that HAS not turned out to be the case. The team came in very, very quickly, it has to be said. (…) And I was SURPRISED that a certain routine was established pretty quickly.” (Doctor_No1_management-level, Pos. 14).

However, difficulties were described, especially assessing psychosocial items and burden of relatives at the beginning of care, and that all professionals have the same understanding of the PCOMs and apply them consistently.

“When two staff members were involved, they had completely different assessments. And I always found this very telling of how difficult/what a learning process it is to have a common assessment. Because that is what we need, a common assessment of some kind, so that we can also assess the process and so that I can say: "Oh, okay, not only because person A saw it that way, then I know, ah, person A always sees it that way", but that we somehow find a common language and, yes, evaluation.” (Doctor_No10, Pos. 36–38).

Furthermore, it was described that in SPHC contact is not as regular as in the inpatient setting; usually situations are dynamic, often change and frequently only snapshots are possible; teams operate as guests in a patient’s home environment and not all patients and relatives understand what SPHC includes, e.g. talking about psychosocial issues.

“I think when I work in the hospital, I have completely different (prerequisites)/I always think when I'm at home, I'm a GUEST. And then I see so MUCH within a very short time, I have such a broad view then. But in the hospital I already have so much structure that I can categorise much more and much faster. And that is simply much more difficult at home.” (Nurse_No17_management-level, Pos. 54–60).

It was described to be challenging to assess the end of care episodes when patients died and professionals were not present. Another criticism of PCOMs was that the individuality of care and the quality of symptoms cannot be represented, and there was concern about loss of time for patients due to the more time-consuming documentation.

“What I'm missing, for example, starting at the top with pain, is that the symptom itself doesn't reflect any quality, yes? So is it tearing now, is it burning now? So I have/must have noted somewhere then, what kind of pain is it right now?” (Nurse_No9, Pos. 36–42).

*Internalisation* and thus the recognition of value took place when users recognised their own/clinical benefit from using PCOMs or not when benefits were not recognised. Aspects mapped in the components *cognitive participation* (*legitimation*) and *collective action* (*interactional workability*) are relevant for this process. However, it may also be possible that meaning was not recognised because of one’s attitude towards palliative care, that it should not be operationalised due to the individuality and therefore text/progress reports were perceived as more important and valuable.

“It is becoming clear that we have not adopted any of this. And so there have been lively discussions here over and over again, (…) because we have just found that it is not individual enough.” (Nurse_No17_management-level, Pos. 18).

#### Cognitive participation

*Cognitive participation* (‘working out participation’) deals with how people participate in order to use PCOMs. *Initiation* is about the role leadership/local facilitators took on, and different approaches could be identified. One approach is characterised by an active role with open communication about expectations and long-term use of PCOMs. The team was involved in decisions, and training in the use of PCOMs was offered during working hours. In these teams coherence at management level was present and well developed.

“And that was actually quite clear to us, everyone should take a look at it. This is working time for all employees. It doesn't matter whether you were on duty on the day of the introduction or not. If you looked at it later, you do that during your working hours, that was PLANNED. And that was certainly very GOOD.“ (Doctor_No10, Pos. 34).

In another team coherence was not well developed at the beginning, but the management level was open to using PCOMs, applied them themselves and initiated discussions about them. In one team, the management level was sceptical and little sense was seen in the application, which was reflected in the fact that there was no open communication, and the team was not involved in decisions. In two teams, management level did not take on a special role.

In terms of *enrolment*, statements about the guidance provided by the research team, training, and training materials were included. Availability and support of the research team was considered positive, as were pocket cards as part of training materials with brief information provided. Manuals were used less because they were considered too extensive. Views on education sessions conducted by the research team were divided, some considered them too theoretical, others good and helpful.

An aspect of *cognitive participation* is the *legitimation* of using PCOMs. This is mainly about personal processes of each individual and how people engage with new things.

“And there, too, it's very different. One person says: "I'll do it once", and immediately says: "Ah, that's all stupid. That is/" Because at first it is of course more difficult when I leave my familiar environment and have to document. The other person might take three, four, five home visits and then says afterwards: "That's good".” (Nurse_No7_management-level, Pos. 18).

Another factor was whether participants recognised their own benefit through using PCOMs, e.g. a quick overview of the situation through a graphical display, focussing holistically on patient/family, or availability of data across settings. The last aspect involves *activation*. All teams discussed whether their measurements were reliable, e.g. how much they differed between professionals. Naming a responsible person as an internal facilitator/champion was particularly helpful and necessary in case of questions or uncertainties.

#### Collective action

*Collective action* (‘doing it’) is concerned with how people integrate PCOMs into their daily work. *Interactional workability* addresses how participants described their use of PCOMs in their everyday work. The predefined structure of PCOMs was used in consultations with patients and relatives, for example in taking the medical history, which was considered to be helpful as all items had to be captured; therefore no important issues were forgotten and all dimensions of care (physical, psychological, social and spiritual) were covered.

“I think that if you use them in a way (…) that they are FUNCTIONAL, no, that you really ask yourself what do I want to KNOW, no, then they are absolutely helpful. So just for the sake of completeness, not to forget anything and also to bring a certain system into history taking.” (Doctor_No16_management-level, Pos. 13–14).

PCOMs led to a content-related discussion in the team about symptoms/problems, actions were derived from it and their effectiveness was evaluated.

“AND THEN we only ever corrected THINGS that had changed. And we also talked about it like this, yes, okay, if we now see a new pain that is now suddenly at FOUR, we have to somehow try to get a grip on it within two or three days. Does that happen?” (Doctor_No11_management-level, Pos. 43–44).

The graphical display was used to get a quick overview of symptoms/problems, which was considered especially helpful during on-call times or to prepare for home visits.

“When I use it, I find it very nice to have this overview. It's almost like a kind of medical chart that opens up afterwards, with the different colours, where I can see at a glance in which areas there are changes, improvements or deteriorations. I think that's very nice. So that gives me an overview and I like to work with that.” (Nurse_No9, Pos. 34).

PCOMs led to the same language being spoken, helped to adopt a common attitude in the team, and documentation could be reduced and simplified. Palliative care phases were used to prioritise care and to get an overview of the situation of all patients. However, there was also feedback from individual participants that PCOMs were not used at all and only assessed for the study. Interviewees mentioned that in terms of *relational integration* using PCOMs created a responsibility for everyone, as colleagues relied on correct measurement.

“And of course you can also transfer the responsibility here in that, as I assess something, the next person who takes over my patients also assumes that this is the assessment of the (deleted—own name) and then it fits.” (Nurse_No17_management-level, Pos. 12).

*Skill-set workability* was fulfilled as in most teams PCOMs were divided according to expertise, for example need for care in everyday life was assessed by nurses. From the interview with the psychologist/social worker it emerged that PCOMs were considered too medical and therefore less suitable for the psychosocial field.

“I have experienced these tools (…) in a way, I was also present at the introduction that it primarily had, I say, medical-nursing aspects. So it was designed more FOR doctors and nurses. That's how I experienced it. (…) And I, as a psychologist, but also within the framework of the psychosocial service, I have social services AND psychological conversations, which I do, I did NOT feel addressed in this way. (Psychosocial_No8, Pos. 4).

Resources were made available in all teams and overtime resulting from using PCOMs was accepted. Nevertheless, individual staff members would have liked more time for familiarisation (*contextual integration*).

#### Reflexive monitoring

How people individually and collectively appraised using PCOMs is considered in the section *reflexive monitoring* (‘reflecting on it’). The first aspect *systematisation* refers to whether participants got insights into the analyses of the collected data material. This was provided by regular feedback and a final report of the COMPANION study, which was evaluated positively. However, it was criticised that a presentation of the situation at beginning and end of care was not helpful, but that the depiction of care trajectories is needed. So far, no further analyses were conducted in any team beyond the study-related period. *Communal appraisal* was predominantly positive. Aspects mentioned were that the focus was directed comprehensively to patients, furthermore to symptom burden and the whole care concept/system including the relatives.

“I/we found it absolutely great that the focus was shifted AWAY from the, let's say, four to six main symptoms of palliative care to a broader spectrum. Because it made us quite often look again at points with the patients or ask questions, investigate, which we didn't have in mind before and maybe wouldn't have grasped at all if we had only had our standard like that. I/we definitely appreciated THAT. And ALSO the focus on psychosocial items was actually by far too neglected in our everyday working life before, I think. (…) And it has often led us in the TEAM, in team meetings, to think about these points again and to look again at where we have a different approach to the patients.” (Doctor_No10, Pos. 18).

Time savings through shortened documentation were described, and that reading progress reports also required less time as a result. In addition, the structure of PCOMs can save time in consultations, so that more time is available for other topics. By using standardised PCOMs, a common and uniform language can be spoken, the same data provided and used across settings, thus avoiding loss of information. Major negative aspects mentioned were that using PCOMs can create an additional time burden and additional work. In the context of *individual appraisal*, it was noted which further benefits the participants could imagine. It was mentioned that PCOMs could serve as screening instruments to identify palliative care needs, to make the activities of SPHC visible, to enable comparisons between services, and to generate transparent data for third parties, such as research or health insurance providers. Furthermore, it was suggested that other people, such as professionals in nursing homes or relatives, should be involved in measuring outcomes. *Reconfiguration* has taken place to varying degrees. Two teams completely changed documentation in line with the study because they considered it to be advantageous. One team has changed documentation due to a decision of the management level and another one integrated palliative care phases into their new electronic patient record. Only one team kept its old documentation, but individuals apply PCOMs they already used before more consistently.

### Implementation outcomes

The last section of the NPT coding manual deals with how things change when interventions are implemented. This cannot be answered on the basis of the data material, as PCOMs have not been used long-term.

## Discussion

In this study, we identified clearly enabling and normalising factors. These were open communication, internal facilitators/champions for application, leadership involvement and management configuration, suitable feedback of the outcome measurements, and better understanding of patients’ and relatives’ needs. In contrast, there were also barriers including the lack of assessing psychosocial aspects, subjectivity of scoring, attitude of individuals, difficulty of use and integration in the electronic documentation system, size and fluctuation of teams, and organisation of care.

Bradshaw et al. reported similar findings in their qualitative study with healthcare professionals in the UK in terms of the subjectivity of scoring, and understanding the benefits of outcome measures. They also emphasized the importance of technical implementation and that data collected is analysed and returned to the teams in an appropriate way [14]. In addition, results are comparable to Sandham et al., who interviewed practitioners in SPHC in New Zealand, and concluded that outcome measures provide structure, lead to a holistic view of the patient, help to focus more on psychosocial needs and to develop a common clinical language [31]. A recent study from Germany by Seipp et al. evaluated the implementation of patient-reported outcome measurement (PROM) in SPHC for care quality evaluation in one federal state. They report that SPHC teams accepted PROMs in their daily working life and recognized the benefit for quality evaluation, but the usefulness of the measurement responses for their practical work was lacking. Furthermore, staff members reported difficulties in assessing PCOMs [32]. The results of our study demonstrate that benefits of using PCOMs in clinical practice are recognised under certain circumstances, e.g. when integrated in existing electronic patient record and graphically displayed. Both studies show that using outcome measures has been established differently in teams, even though they all provide SPHC—suggesting that in some teams it might be easier to implement outcome measurement than in others. The individuality of teams, their organisation of work, and context in everyday life play a major role, which is in line with Seipp et al. as they emphasise the importance of taking individual team characteristics into account.

PCOC in Australia, in which 177 (an estimated 90%) palliative care services voluntarily participated in 2021 [33], developed a three-stage implementation guide to address challenges in terms of implementation. First, the readiness of the institution/team is checked by assessing the readiness containing requirements for participation, including definition of procedures, roles, and responsibilities. Based on the assessment, an implementation plan is developed including the support of senior executive staff, the selection of champions to facilitate local implementation and work with PCOC Facilitators (e.g. facilitators employed by PCOC and external to the organisation) and to develop workflows including communication processes in teams. As the guide is designed for leaders, it is apparent that they have a special responsibility, which is in line with the results of our study as well as other aspects mentioned. The second stage towards implementation and standardised use can only be started when all requirements in the readiness assessment have been completely fulfilled [34].

Demonstrating patient-centred outcomes will become increasingly important in the future, and required by funding agencies in Germany in the long term. Considering that applying PCOMs has clinical benefits and collecting meaningful and reliable data is a prerequisite for the application of casemix classifications, tailored implementation strategies that break down existing barriers and foster facilitating factors should be developed. A particular challenge will be to implement PCOMs in teams, whose working environment (context) appears less suitable for application. Therefore, it might be helpful to bring them together with teams where the application works well and is beneficial, to learn from each other. Building coherence is paramount and thus understanding the meaningfulness and recognising advantages for individuals and the team. This is not only related to direct patient care and cooperation but also in a broader sense to generate data to demonstrate the effectiveness of palliative care and serve quality assurance. For this purpose, structures need to be developed that the collected data can be analysed in a standardised way and thus made usable for the teams.

Based on the knowledge and consideration of static qualities of the context and their determinants to successful implementation, the Consolidated Framework for Implementation Research (CFIR) might be helpful in a next step to address these challenges. Schroeder et al. have shown that the interplay of NPT (including dynamic implementation processes) and CFIR (including static qualities) is helpful in understanding and clarifying how innovations find their way into routine practice [35].

### Strengths and limitations of the study

A strength of the study is the inclusion of views of different professional groups at both management and non-management levels in five SPHC teams across five states in Germany. A weakness is the limited availability of professionals with psychosocial background with only one interview. However, many SPHC teams are only working with two professional groups (restricted by German regulation for SPHC) and the results therefore reflect the care structures in Germany. Another potential weakness is an unbalanced gender ratio. Nevertheless, we assume that this was compensated by the variance of the other sampling criteria. Furthermore, the COMPANION study might have influenced using PCOMs in clinical practice. However, we believe that this did not influence the results of this study, as difficulties in the application of the outcome measures were mentioned and criticism about the use was also voiced. To minimise socially desirable responses (measurement bias), the interviews in the SPHC teams that were supervised by ELE in the COMPANION data collection were conducted by FH. Lastly, the pandemic may have led to a different perception of the application of PCOMs in clinical practice and this may have had an influence on the response behaviour of interviewees. Since the results correspond with other studies, we do not assume that this is the case. In addition, not all interviews could be conducted shortly in time after the end of the COMPANION data collection due to the pandemic; however, we did not observe any effects on response behaviour and expect that this led to interviewees having more time to gain further experiences.

## Conclusion

Benefits were partly realised by teams, and not all continued to use PCOMs standardised. For those that continued, social (inter-)actions and attitudes among team members and leadership were decisive. The context of teams’ further influenced successful use. Future implementation strategies should especially consider the integration of PCOMs in existing electronic patient records, education and training sessions that support coherence-building (recognition of benefits), internal facilitators/local champions, and ensuring frequent analyses of collected data as it is beneficial for the teams.

### Supplementary Information

**Additional file 1. **COREQ Reporting Checklist.

**Additional file 2. **Patient-centred outcome measures.

**Additional file 3. **Sampling frame.

**Additional file 4. **Interview guide.

**Additional file 5. **Coding tree.

## Data Availability

The datasets generated and/or analysed during the current study are not publicly available due [data agreement reasons: interview manuscripts cannot be anonymised entirely, the interviewees’ anonymity would not be guaranteed] but are available from the corresponding author on reasonable request.

## Abbreviations

PROMs: Patient-reported outcome measures
PCOMs: Patient-centred outcome measures
NPT: Normalization Process Theory
SPHC: Specialist palliative home care
PCOC: Palliative Care Outcome Collaboration
OACC: Outcome Assessment and Complexity Collaboration
CMO: Context-Mechanism-Outcome
COREQ: COnsolidated criteria for REporting Qualitative research
PROM: Patient-reported outcome measurement
CFIR: Consolidated Framework for Implementation Research
