# Supplementary material for: Using normalization process theory to evaluate the use of patient-centred outcome measures in specialist palliative home care—a qualitative interview study
Source: BMC Palliat Care. 2024 Jan 3;23:1. doi: 10.1186/s12904-023-01329-8 (PMC10763078; doi:10.1186/s12904-023-01329-8)
Supplement: Supplementary file 1 — Additional file 1. COREQ Reporting Checklist. [file 12904_2023_1329_MOESM1_ESM.docx]

**Additional file 1.** COREQ Reporting Checklist

| Domain 1: Research team and reflexivity | |
| --- | --- |
| Personal Characteristics | |
| 1. Interviewer/facilitator | Eva Lehmann-Emele (ELE) and Farina Hodiamont (FH) conducted the interviews. |
| 1. Credentials | ELE - Master of Science, FH - Dr. rer. biol. hum. |
| 1. Occupation | Research Associate |
| 1. Gender | Female |
| 1. Experience and training | Not reported due to space limitations: EL - Master Degree in Health Service Research, research experience in the context of qualitative studies and one quantitative study, FH - Master Degree in Sociology, conception and realization of several qualitative and quantitative studies. |
| Relationship with participants | |
| 1. Relationship established | Not reported due to space limitations: The research team and some interviewees did know each other, but there was no established relationship. |
| 1. Participant knowledge of the interviewer | Not reported due to space limitations: The interviewees did only know that the research team is conducting research on the topic. |
| 1. Interviewer Characteristics | Not reported due to space limitations: The interviewers have a research interest in outcome measurement and complexity in specialist palliative care and therefore, the implementation of outcome measurement tools in German clinical practice is relevant. |
| Domain 2: Study design | |
| Theoretical Framework | |
| 1. Methodological orientation and theory | Stated in the methods sections ‘design’ and ‘data management and analysis’. Data was analysed using Framework method and contextualised within Normalization Process Theory. |
| Participant selection | |
| 1. Sampling | Stated in the methods section, ‘setting and participants’, purposive and convenient sampling. |
| 1. Method of approach | Stated in the methods section, ‘setting and participants’, an invitation letter to take part in the interview study was sent by email by ELE or they were reached by phone. |
| 1. Sample size | Reported in the first paragraph of the results section and Table 2. |
| 1. Non-participation | Reported in the first paragraph of the results section. |
| Setting | |
| 1. Setting of data collection | Reported in the methods section ‘setting and participants’. |
| 1. Presence of non-participants | Not reported due to space limitations: No one else was present besides the participants and the interviewer. |
| 1. Description of Sample | Reported in the first paragraph of the results section and Table 2. |
| Data Collection | |
| 1. Interview Guideline | The development of the interview guide is described in the methods section, ‘Interview guide’. The translated interview guide was added as an additional file. The original interview guide (in German language) is available from the authors on reasonable request. |
| 1. Repeat interviews | Not reported due to space limitations: No repeat interview was necessary. Interviewees were asked to be contacted again in case of any uncertainties or lack of information becoming evident subsequent to the interview. The project team did not have to make use of this possibility. No technical problems occurred which could have prompted the necessity of a repeat interview. |
| 1. Audio/Visual recording | Reported in the methods section, “data management and analysis”. |
| 1. Field notes | Not reported due to space limitations: A field-note form was filled in by the researchers after each interview, covering the following topics: **Expert-interviewer relationship** (🡪First impression, did they already know each other, etc.?), **Interview setting, incidents/interruptions and atmosphere** (🡪Were there any disturbing factors (interruptions, noise, light, temperature)?), **Perceived moods of the interviewee** (🡪 e.g. emotional, upset, defensive), **Difficulties in conducting the interview**, **Comments on content** (🡪 Did topics come up that were not covered by the interview guide? Was there a theme that dominated the interview?), **Feelings of the interviewer** (🡪 How did the interviewer experience the atmosphere, what felt good, what did not feel good? Were there insecurities, discomfort, etc.?) |
| 1. Duration | Reported in the first paragraph of the results section. |
| 1. Data saturation | Reported in the first part of the results section. |
| 1. Transcripts returned | Not reported due to space limitations: Transcripts were not returned to participants. Statements and descriptions were, however, continuously confirmed by the interviewing researcher during the interview in order to guarantee the correct understanding. |
| Domain 3: Analysis and findings | |
| Data analysis | |
| 1. Number of data coders | Reported in the methods section ‘data management and analysis’. |
| 1. Description of the coding tree | Only by representing the findings, due to space limitations. Available from the authors on reasonable request. |
| 1. Derivation of themes | Reported in the methods section ‘data management and analysis’. |
| 1. Software | Reported in the methods section ‘data management and analysis’: MaxQDA 2022. |
| 1. Participant checking | Not reported due to space limitations: Participants were not asked to provide feedback on the findings. |
| Reporting | |
| 1. Quotations presented | Quotations are presented and identified, reported in results section. |
| 1. Data and findings consistent | Yes |
| 1. Clarity of major themes | Yes, we present the major themes. |
| 1. Clarity of minor themes | Yes, as far as space limitations permitted, we discuss minor themes, too. |
