# Supplementary material for: Using normalization process theory to evaluate the use of patient-centred outcome measures in specialist palliative home care—a qualitative interview study
Source: BMC Palliat Care. 2024 Jan 3;23:1. doi: 10.1186/s12904-023-01329-8 (PMC10763078; doi:10.1186/s12904-023-01329-8)
Supplement: Supplementary file 2 — Additional file 2. Patient-centred outcome measures. [file 12904_2023_1329_MOESM2_ESM.docx]

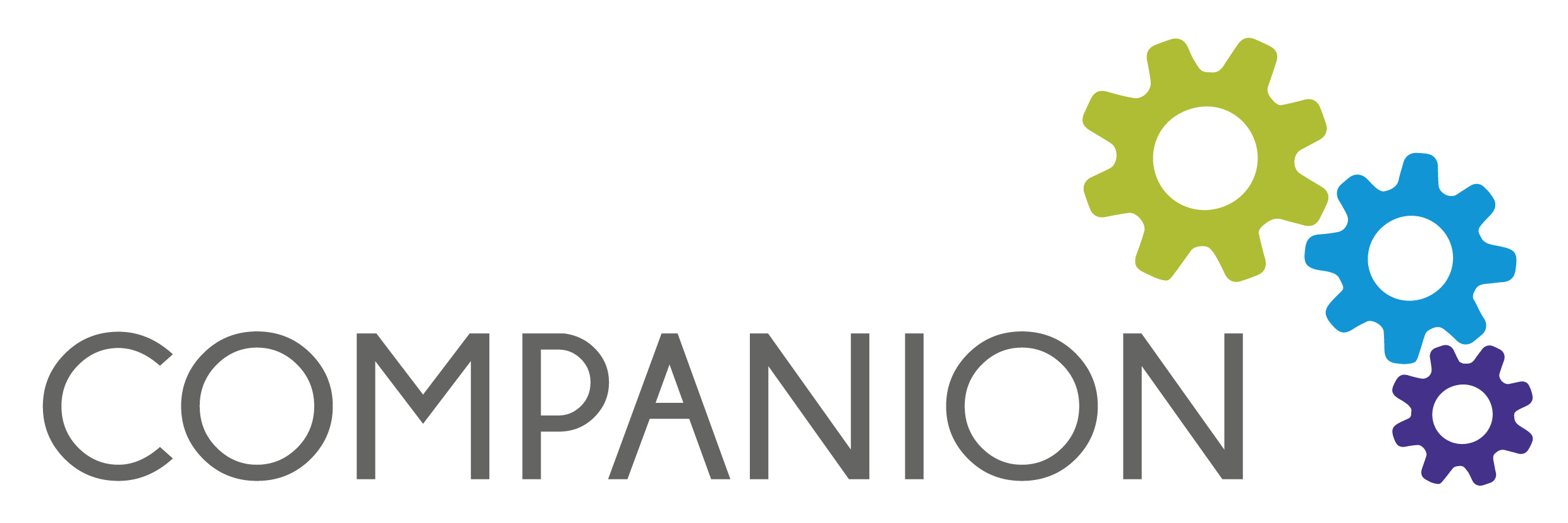
**Additional file 2.** Patient-centred outcome measures

| **Integrated Palliative Care Outcome Scale - IPOS** | | | | | | |
| --- | --- | --- | --- | --- | --- | --- |
| **How much is the patient affected by the following symptoms/problems?** | | | | | | |
|  | *Not at all* | *Slightly* | *Moderatly* | *Severly* | *Overwhelmingly* | *Not assessable* |
| Pain |  |  |  |  |  |  |
| Shortness of breath |  |  |  |  |  |  |
| Weakness or lack of energy |  |  |  |  |  |  |
| Nausea |  |  |  |  |  |  |
| Vomiting |  |  |  |  |  |  |
| Poor appetite |  |  |  |  |  |  |
| Constipation |  |  |  |  |  |  |
| Sore or dry mouth |  |  |  |  |  |  |
| Drowsiness |  |  |  |  |  |  |
| Poor mobility |  |  |  |  |  |  |
|  | *Not at all* | *Occasionally* | *Sometimes* | *Most of the time* | *Always* | *Not assessable* |
| Patient worried/anxious |  |  |  |  |  |  |
| Family worried/anxious |  |  |  |  |  |  |
| Patient depressed |  |  |  |  |  |  |
|  | *Always* | *Most of the time* | *Sometimes* | *Occasionally* | *Not at all* | *Not assessable* |
| Patient at peace |  |  |  |  |  |  |
| Patient able to share feelings |  |  |  |  |  |  |
| Getting information |  |  |  |  |  |  |
|  | *Problems addressed/No problems* | *Problems mostly addressed* | *Problems partly addressed* | *Problems hardly addressed* | *Problems not addressed* | *Not assessable* |
| Problems addressed |  |  |  |  |  |  |
|  |  |  |  |  |  |  |
|  |  |  |  |  |  |  |
| **Palliative Care Problem Severity- PCPSS** | | | | | | |
| **How severe are the following symptoms or problems?** | | | | | | |
|  | *Absent* | *Mild* | *Moderate* | *Severe* |  |  |
| Pain |  |  |  |  |  |  |
| Other symptoms |  |  |  |  |  |  |
| Psychological/spiritual |  |  |  |  |  |  |
| Family/Carer |  |  |  |  |  |  |
| Confusion |  |  |  |  |  |  |
| Restlessness |  |  |  |  |  |  |
| **Australian-modified Karnofsky Performance Status - AKPS** | | | | | | |
| **Current functional status of the patient** | | | | | | |
| Normal; no complaints; no evidence of disease | | | | | 100 |  |
| Able to carry on normal activity; minor sign of symptoms of disease | | | | | 90 |  |
| Normal activity with effort; some signs or symptoms of disease | | | | | 80 |  |
| Cares for self; unable to carry on normal activity or to do active work | | | | | 70 |  |
| Able to care for most needs; but requires occasional assistance | | | | | 60 |  |
| Considerable assistance and frequent medical care required | | | | | 50 |  |
| In bed more than 50% of the time | | | | | 40 |  |
| Almost completely bedfast | | | | | 30 |  |
| Totally bedfast and requiring extensive nursing care by professionals and/or family | | | | | 20 |  |
| Comatose or barely rousable | | | | | 10 |  |
| Dead | | | | | 0 |  |
|  |  |  |  |  |  |  |
|  |  |  |  |  |  |  |
| **Barthel-Index** | | | | | | |
| **Patient's daily ability (the higher the value, the more independent)** | | | | | | |
| 1. Bowels | | Continent | | | | 2 |
|  |  | Occasional accident | | | | 1 |
|  |  | Incontinent (or needs to be given enemata) | | | | 0 |
| 2. Bladder | | Continent (for over 7 days) | | | | 2 |
|  |  | Occasional accident | | | | 1 |
|  |  | Incontinent or catheterized | | | | 0 |
| 3. Grooming | | Independent | | | | 1 |
|  |  | Needs help with personal care | | | | 0 |
| 4. Toilet Use | | Independent | | | | 2 |
|  |  | Needs some help | | | | 1 |
|  |  | Dependent | | | | 0 |
| 5. Feeding | | Independent | | | | 2 |
|  |  | Needs some help | | | | 1 |
|  |  | Unable | | | | 0 |
| 6. Transfers (bed to chair and back) | | Independent (including wheelchair) | | | | 3 |
|  |  | Minor help | | | | 2 |
|  |  | Major help | | | | 1 |
|  |  | Unable | | | | 0 |
| 7. Mobility (on level surface) | | Independent (but may use any aid, e.g. stick) | | | | 3 |
|  |  | Walks with help of one person | | | | 2 |
|  |  | Wheelchair independent | | | | 1 |
|  |  | Immobile | | | | 0 |
| 8. Dressing | | Independent | | | | 2 |
|  |  | Needs help | | | | 1 |
|  |  | Dependent | | | | 0 |
| 9. Stairs | | Independent up and down | | | | 2 |
|  |  | Needs help | | | | 1 |
|  |  | Unable | | | | 0 |
| 10. Bathing | | Independent | | | | 1 |
|  |  | Dependent | | | | 0 |
|  |  |  |  |  |  |  |
|  |  |  |  |  |  |  |
| **Palliative Care Phase** | | | | | | |
| **Palliative care phase of the patient?** | | | | | | |
| - Symptoms and problems (psychosocial, spiritual) adequately controlled by care plan. - Situation of family caregivers relatively stable, no new problems apparent. | | | | | Stable | |
| - Urgent change in care plan or emergency intervention required because of new unexpected symptom/problem or unexpected rapid worsening of an existing symptom/problem. | | | | | Unstable | |
| - Care plan takes into account expected symptoms/problems, regular review required. | | | | | Deteriorating | |
| - Death is likely within days. | | | | | Terminal | |
| - Patient has died. | | | | | Bereavement | |
