# Supplementary material for: Using normalization process theory to evaluate the use of patient-centred outcome measures in specialist palliative home care—a qualitative interview study
Source: BMC Palliat Care. 2024 Jan 3;23:1. doi: 10.1186/s12904-023-01329-8 (PMC10763078; doi:10.1186/s12904-023-01329-8)
Supplement: Supplementary file 3 — Additional file 3. Sampling frame. [file 12904_2023_1329_MOESM3_ESM.docx]

**Additional file 3.** Sampling frame

| **Characteristics** | | **n/Team** |
| --- | --- | --- |
| Profession | Doctors | 1 |
|  | Nurses | 1 |
|  | Allied health professionals (psychologists, social workers) | 0-3 |
| Position | Management level  Non-management level | 1-2  1-3 |
| Work experience (years) | < 5  5 - 10  > 10 | 0-5  0-5  0-5 |
| Age (years) | < 39  40-55  > 55 | 0-5  0-5  0-5 |
| Sex | Female  Male  Other | 0-4  Min. 1  0-4 |
| Total |  | 12-30 |
