# Supplementary material for: Using normalization process theory to evaluate the use of patient-centred outcome measures in specialist palliative home care—a qualitative interview study
Source: BMC Palliat Care. 2024 Jan 3;23:1. doi: 10.1186/s12904-023-01329-8 (PMC10763078; doi:10.1186/s12904-023-01329-8)
Supplement: Supplementary file 4 — Additional file 4. Interview guide. [file 12904_2023_1329_MOESM4_ESM.docx]

**Additional file 4.** Interview guide

1. Clarification about the study and contents of Informed Consent

*To get started:*

Some time ago, you successfully completed the data collection of the COMPANION project and were trained in the use of clinical assessment instruments (IPOS, PCPSS, etc.). During the interview, I would like to talk about your experiences with the use of these assessment instruments. You are welcome to tell me your own opinion or to include the voices/opinions of your team.

Collect socio-demographic data and indicate that I am taking notes.

1. Open conversation

******Start of audio recording******

| **Part I – Experience with clinical assessment tools prior to participation in the COMPANION project** | | |
| --- | --- | --- |
| Key questions | In-depth questions | Sustainment questions |
| 🡪 At the beginning of the interview, I would ask you to recall the time before the introduction of the assessment tools and data collection. My first question would be:   - What experiences have you had with assessment tools so far, independently of the COMPANION project? | - Which assessment tools did you already know? - How did you use them? - What was it used for and what did it look like in everyday working life? - What did you think about assessment tools and their application? - Can you please give me an example? | - What are you thinking about right now? - I noticed that you hesitated a moment ago. What was the reason for that? - Could you describe it in more detail? - Could you elaborate on that? - Could you be more specific about that, please? - What exactly do you mean by that? - Could you please explain your statement a little bit more? |

| **Part II - Experiencing the use of the assessment tools during data collection in the COMPANION project** | | |
| --- | --- | --- |
| Key questions | In-depth questions | Sustainment questions |
| 🡪 In the second part, I would like to talk about your experiences with the assessment tools during the COMPANION project:   - How did you experience the use of assessment tools in the project? | - What did you like, what not so much? - Could you please give me some examples? | - What are you thinking about right now? - I noticed that you hesitated a moment ago. What was the reason for that? - Could you describe it in more detail? - Could you elaborate on that? - Could you be more specific about that, please? - What exactly do you mean by that? - Could you please explain your statement a little bit more? |
| - Did the use of the assessment tools have an impact on your everyday professional life? - Has anything changed in your team as a result? | - If yes, which/what? - Could you please describe this in more detail? Give examples? |  |
| - How did you experience the training and feedback sessions? - How would you rate the training material? (Manuals) | - What else would you have wished? |  |
| - To what extent has your pre-existing opinion about assessment tools changed because of the project? | - What do you think is the reason for that? - Could you please describe it in more detail? |  |

| **Part III - Further implementation of the assessment tools after the end of the data collection of the COMPANION project** | | |
| --- | --- | --- |
| Key questions | In-depth questions | Sustainment questions |
| 🡪 In the third and last part of the interview, I would be interested to know to what extent the assessments tools are still part of your everyday work:   - Are they still applied? | | - What are you thinking about right now? - I noticed that you hesitated a moment ago. What was the reason for that? - Could you describe it in more detail? - Could you elaborate on that? - Could you be more specific about that, please? - What exactly do you mean by that? - Could you please explain your statement a little bit more? |
| 🡪 If **yes**: Which assessment tools are still used? | |  |
| - What made you/the team decide to continue using them? | - Können Sie mir das bitte genauer erklären/ein Beispiel geben? |  |
| - How do you use the assessment tools in everyday life? Are they mentioned, for example, in the handover or in team meetings? | - What does this look like in practice? - Who assesses the assessments and how often? - Where and how is this documented/recorded? - To what extent do you derive actions from this? |  |
| - What challenges did you face? | - How were they solved? - What or who was helpful? |  |
| - What happens to the data collected through the assessment tools? | - Do you have access to it? - Will the data be evaluated/used? - Are there plans to do so? |  |
| - Detached from your everyday work, do you see any further benefit in using the assessment tools? | - Can you please give me examples of this? |  |

| 🡪 If **no**: Why not? | | - What are you thinking about right now? - I noticed that you hesitated a moment ago. What was the reason for that? - Could you describe it in more detail? - Could you elaborate on that? - Could you be more specific about that, please? - What exactly do you mean by that? - Could you please explain your statement a little bit more? |
| --- | --- | --- |
| - What would you or the team have needed to make the use beneficial? | - Who or what could support you/the team? - Could you please give me an example? |  |
| - To what extent do you think the use of assessment tools could be changed or adapted to make their use more acceptable or relevant to your everyday work? | - Could you please give me an example? - Who or what could support you/the team? |  |
| - What do you think about the use of assessment tools in specialist palliative home care? | - Could you please describe in more detail why? |  |
| - Are there any assessment tools that you consider more or less relevant? | - What are the reasons for this? - Could you please give me an example? |  |
| - Do you see a benefit in the use of assessment tools independent of your daily work in specialist palliative home care? | - Could you please describe it in more detail why? |  |

*At the end:*

- Is there anything we haven't touched on yet that you would like to share with me?

Otherwise, thank you very much for taking part in the interview!

******Stop audio recording******
