# Supplementary material for: Using normalization process theory to evaluate the use of patient-centred outcome measures in specialist palliative home care—a qualitative interview study
Source: BMC Palliat Care. 2024 Jan 3;23:1. doi: 10.1186/s12904-023-01329-8 (PMC10763078; doi:10.1186/s12904-023-01329-8)
Supplement: Supplementary file 5 — Additional file 5. Coding tree. [file 12904_2023_1329_MOESM5_ESM.docx]

**Additional file 5.** Coding tree

| **Coding tree** | |
| --- | --- |
| Domain according to Coding Manual by May et al. (2022) | Subcodes developed out of the interview data material |
| **IMPLEMENTATION CONTEXT - How is the intervention (using PCOMs) influenced by the environment?** | |
| **Strategic intentions**  How does the working environment affect the design and planning of the use of PCOMs? | Only study participation planned or further implementation of PCOMs |
| **Adaptive execution**  How does the working environment affect the way in which users can find and implement ways of working that make the use of PCOMs an implementable project for practice? | Other software provider than used in the COMPANION study |
|  | Challenges with electronic documentation |
| **Negotiating capacity** How does the context affect the extent to which the use of PCOMs can fit or be integrated into the existing ways of working of their users? | Reference person care system or changing patients and relatives |
|  | Size & consistency/characteristics of the team |
|  | Types of care provided by the teams (range of services) |
|  | Structure of handovers (face-to-face or digital/text-based) |
|  | Independent team or affiliated with a clinic |
|  | Hierarchical structures between management and non-management or professional groups |
| **Reframing organisational logistics** How do the existing social-structural and social-cognitive resources affect the implementation environment? | Attitude/coherence of the management level/key persons |
| **IMPLEMENTATION MECHANISMS - What is the actual work to do in realising the intervention (using PCOMs)?** | |
| **Coherence – ‘making sense of it’** | |
| **Differentiation**  How do participants distinguish the use of PCOMs from their previous way of working? | See previous experience |
|  | Documentation in text/progress reports |
| **Communal and individual specification**  How do participants collectively and individually achieve an understanding about the use of PCOMs? | Previous experiences |
|  | Unfamiliar at first, then quickly found your feet |
|  | Assessing is daily work |
|  | Developing new routines takes time |
|  | Difficult: assessing psychosocial items & relatives  🡪 helpful: intuitive action/assessment |
|  | Difficult: common/same understanding and application of PCOMs |
|  | Difficult: special circumstances in the community setting  🡪 only snapshots/no regularity  🡪 own role in SPHC, acting as a guest/counselling in the home environment of patients and relatives  🡪 assessment at the end of episodes in the event of death |
|  | Difficult: Individuality & quality (of symptoms/care) cannot be depicted by PCOMs alone |
|  | Difficult: Concern about loss of patient time |
| **Internalisation**  Does the use of PCOMs make sense for the people involved? | Purpose is recognised due to own/clinical benefit |
|  | Purpose is not recognised because no personal/clinical benefit is recognised |
|  | Purpose is not recognised (due to attitude & perceived importance of text/progress reports) |
| **Cognitive participation – ‘working out participation’** | |
| **Initiation**  Which role does the leadership/key persons take on? | Positive/coherence pronounced/active |
|  | Critical/coherence less pronounced/passive |
|  | Critical/coherence developed/active |
|  | No special role |
| **Enrolment**  How do participants assess the introduction to the study and the training material and how was it used? | Support from the research team |
|  | Trainings |
|  | Training material |
| **Legitimation**  How do participants come to believe that using PCOMs is right and should be part of their work? | Depending on the person/type, how users engage with new things  🡪 Differences also between the professional groups |
|  | Recognising own/practical advantages increases readiness |
| **Activation**  How do people involved support the use of PCOMs? | Communication within the team/ with colleagues |
|  | Responsible person in and from the practice |
| **Collective action – ‘doing it‘** | |
| **Interactional workability**  How do participants use PCOMs in their everyday work? | Structure of PCOMs is used in discussions with patients & relatives |
|  | Exchange within the (inter-) professional team by evaluating the PCOMs |
|  | Derivation of actions/measures (IPOS/Barthel Index) |
|  | Graphical representation (mouseover) is used |
|  | Documentation is streamlined & simplified |
|  | Palliative care phase is used as a measure for prioritisation & overview of the overall care situation |
|  | Speak a common language/adopt a common attitude |
|  | No use in everyday work |
| **Relational integration**  How does the use of PCOMs affect the trust that participants have in each other? | Responsibility for accurate use/assessment of PCOMs |
| **Skill-set workability**  Is the work required to apply PCOMs allocated appropriately to those involved? | Breakdown of individual PCOMs by professional group |
|  | Social work/psychology assessments not suitable |
| **Contextual integration**  Are resources made available for implementing the use of PCOMs? | Provision of resources |
| **Reflexive monitoring – ‘reflecting on it’** | |
| **Systematisation**  How do those involved have access to information about the impact of using PCOMs? | Feedback on the analysed data (feedback) |
|  | No (knowledge about) further use/analysis of the data |
| **Communal appraisal**  How do participants evaluate the impact of using PCOMs? | Direct all-encompassing focus on patient |
|  | Direct focus on symptom burden |
|  | Focus on care concept/system (palliative care phases) |
|  | Cross-setting data |
|  | Shortened documentation (time) & less text/progress reports |
|  | Time saving through using the structure of PCOMs in conversations |
|  | Additional (time-related) workload/extra effort  🡪 Concern about loss of patient time due to documentation |
|  | Development of a common/standardised language |
| **Individual appraisal**   What further benefits/use of PCOMs can participants envision? | Transparent data for third parties (research/health insurances) |
|  | Ability to show/visualise SPHC activities/tasks, quality assurance, billing |
|  | Screening tool for palliative care needs |
|  | Palliative care phases to argue in favour of earlier inclusion of SPHC |
|  | Assessment of PCOMs by relatives/staff in nursing homes |
| **Reconfiguration**  How do practitioners change their own work in response to their appraisal of using PCOMs? | Already used outcome measure are used more consistently |
|  | Further use of PCOMs similar to the study |
|  | Adaptation of own documentation system for further use |
|  | Integration of palliative care phases into own documentation system |
|  | No further use |
| **OUTCOME – How do things change when the intervention (using PCOMs) is implemented?** | |
| **Intervention performance** What practices have changed over time through the operationalisation, implementation and reproduction of the use of PCOMs? | No findings - PCOMs have not been used long-term. |
| **Relational restructuring** In what ways has the use of PCOMs changed the way people are organised and relate to each other? | No findings - PCOMs have not been used long-term. |
| **Normative restructuring**  In which way has the use of PCOMs changed the norms, rules and resources that govern action? | No findings - PCOMs have not been used long-term. |
| **Sustainment (normalisation)**  In what way has the use of PCOMs become established in practice? | No findings - PCOMs have not been used long-term. |
